# Supplementary material for: Foundational Aspects for Incorporating Dependencies in Copula-Based Bayesian Networks Using Structured Expert Judgments, Exemplified by the Ice Sheet–Sea Level Rise Elicitation
Source: Entropy (Basel). 2024 Nov 5;26(11):949. doi: 10.3390/e26110949 (PMC11592448; doi:10.3390/e26110949)
Supplement: Supplementary file 1 [file entropy-26-00949-s001.zip › Supplementary Materials.pdf]

Supplementary Materials: Foundational Aspects for Incorporating Dependencies in Copula-Based Bayesian Networks Using Structured Expert Judgments, Exemplified by the Ice Sheet–Sea Level Rise Elicitation

Dorota Kurowicka, Willy Aspinall and Roger Cooke

In this supplementary material we provide additional context to the sea level rise (SLR) projections obtained using the advanced Pair Copula Bayesian Network (PCBN) analyses described in our main paper. Basic comparisons with the SLR projections reported by Bamber et al. [1] are presented.

The impact of our dependencies analysis relative to the original Bamber et al.[1] SLR results is summarised in Figure S1, which shows an exceedance probability chart for projected sea level rise by 2100CE under a +5°C global mean surface temperature rise.

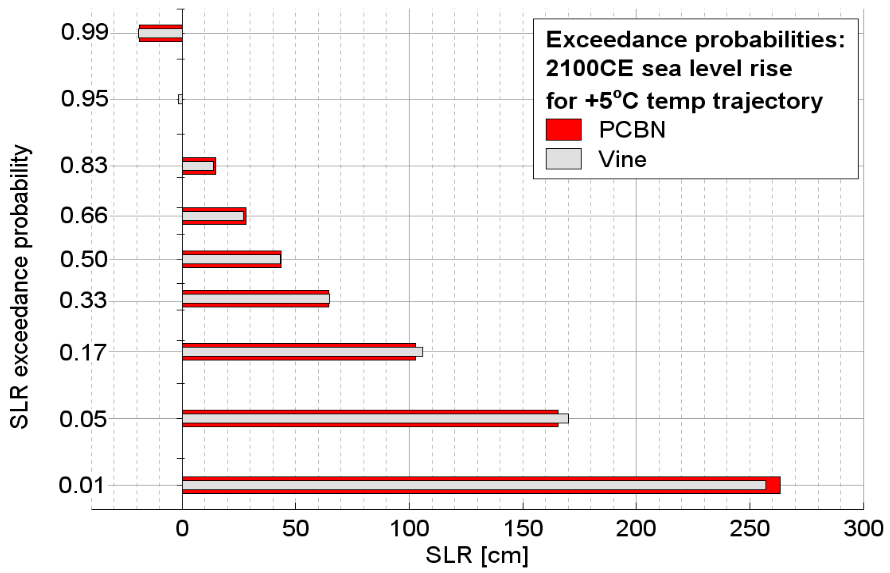

Figure S1. Sea level rise (SLR) exceedance probability chart: Bamber et al.[1] vine results are shown as grey bars, and exceedance probabilities obtained from our updated PCBN analysis are shown as red bars.

On the basis of this chart, three remarks can be offered. For high (from p = 0.99 to p = 0.33) probabilities of exceedance, the differences between the two analyses (i.e., the original vine copula analysis and the updated PCBN analysis) are negligible. However, for probabilities of p = 0.17 and p = 0.05, the Bamber et al. results appear fractionally more conservative than our updated estimates, but only by about + 5 cm of SLR. When it comes to the most extreme exceedance probability plotted here, p = 0.01, our PCBN SLR projection is 264 cm SLR by 2100CE, where Bamber et al. reported 257 cm. Again, the numerical differences are not significant.

However, the key point here is that until our more comprehensive dependence uncertainty analysis was performed, it could not be known just how substantial any differences might be. In this particular case they are not substantial but, when designing mitigation measures to protect against a low-probability high-consequence extreme event in other

circumstances, a 10% increase in hazard could be very costly (e.g., building a sea wall to defend an existing nuclear power plant through to 2100CE and beyond).

We can take the analysis of our model results, in comparison with the earlier Bamber et al. estimates, one step further, as follows (Figure S2).

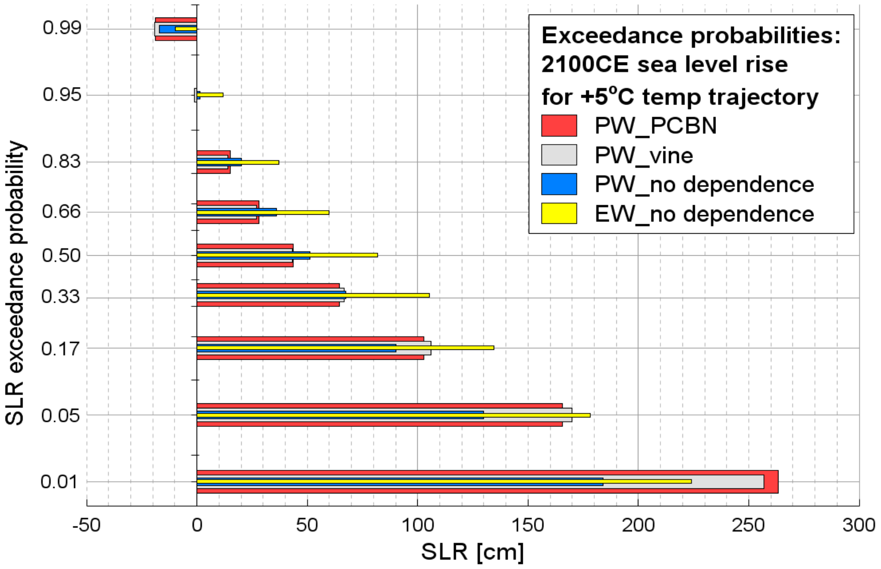

Figure S2. This chart shows SLR exceedance probabilities for four modelling cases: PW_PCBN (red bars) is our updated Pair Copula Bayesian Network analysis ; PW_vine (grey bars) were the equivalent exceedance probabilities for the original Bamber et al. (2019) vine analysis; PW_no dependence (blue bars) show SLR exceedance probabilities assuming ice sheet processes all act independently (see main text); EW_no dependence (yellow bars) represent SLR exceedance probabilities using an equal weights combination of expert judgements with the assumption ice sheet processes act independently.

For nearly all the exceedance probabilities shown in Figure S2, those for equal weights (no dependence) SLR estimates (yellow bars) are notably more conservative (i.e., higher SLR) than their performance weights counterparts (blue) or the SLR estimates of the dependence-based modelling cases. But, for the extreme low probability p = 0.01 scenario, the process-independent cases produce substantially lower SLR estimates than those obtained when vine-modelled or PCBN dependencies are included in the analysis.

The reader is referred to Bamber et al. [1] Supplementary Information for further material.

These differences and insights only become evident when a detailed dependence analysis is undertaken; under some conditions, gross discrepancies can arise in projected hazard levels if such inter-relationships between parameters or processes are ignored.

References

1. Bamber, J.L.; Oppenheimer, M.; Kopp, R.E.; Aspinall, W.; Cooke, R.M. Ice sheet contributions to future sea level rise from structured expert judgement. *PNAS* **2019**. https://doi.org/10.5523/bris.23k1jbtan6sjv2huakf63cqgav.
