# Supplementary figures and images for: Foundational Aspects for Incorporating Dependencies in Copula-Based Bayesian Networks Using Structured Expert Judgments, Exemplified by the Ice Sheet–Sea Level Rise Elicitation
Source: Entropy (Basel). 2024 Nov 5;26(11):949. doi: 10.3390/e26110949 (PMC11592448; doi:10.3390/e26110949)

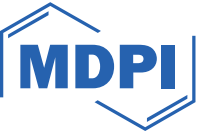

Supplement: Supplementary file 1 [file entropy-26-00949-s001.zip › Definitions/logo-mdpi-eps-converted-to.pdf]

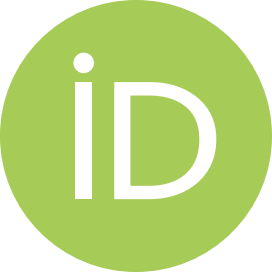

Supplement: Supplementary file 1 [file entropy-26-00949-s001.zip › Definitions/logo-orcid.pdf]

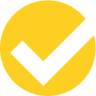

check for  
updates

Supplement: Supplementary file 1 [file entropy-26-00949-s001.zip › Definitions/logo-updates-eps-converted-to.pdf]

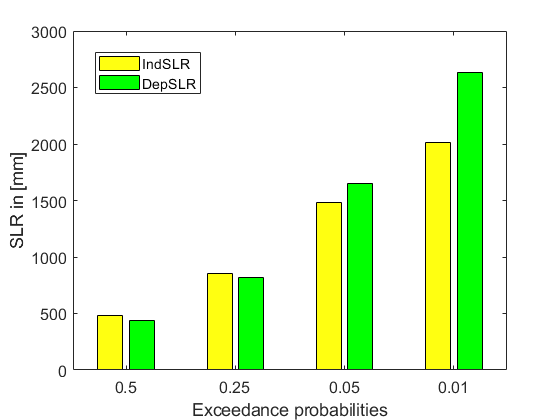

Supplement: Supplementary file 1 [file entropy-26-00949-s001.zip › Figures/ExProb.png]

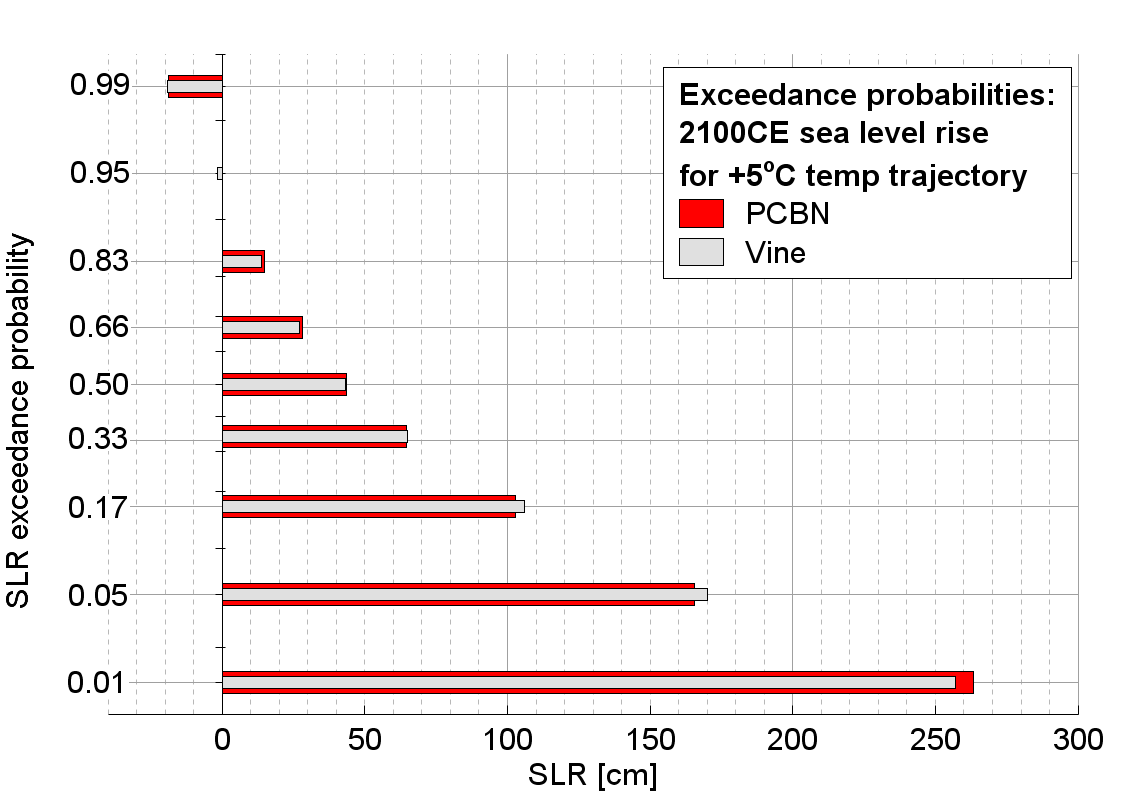

Supplement: Supplementary file 1 [file entropy-26-00949-s001.zip › Figures/Kurowicka SI Fig1.png]

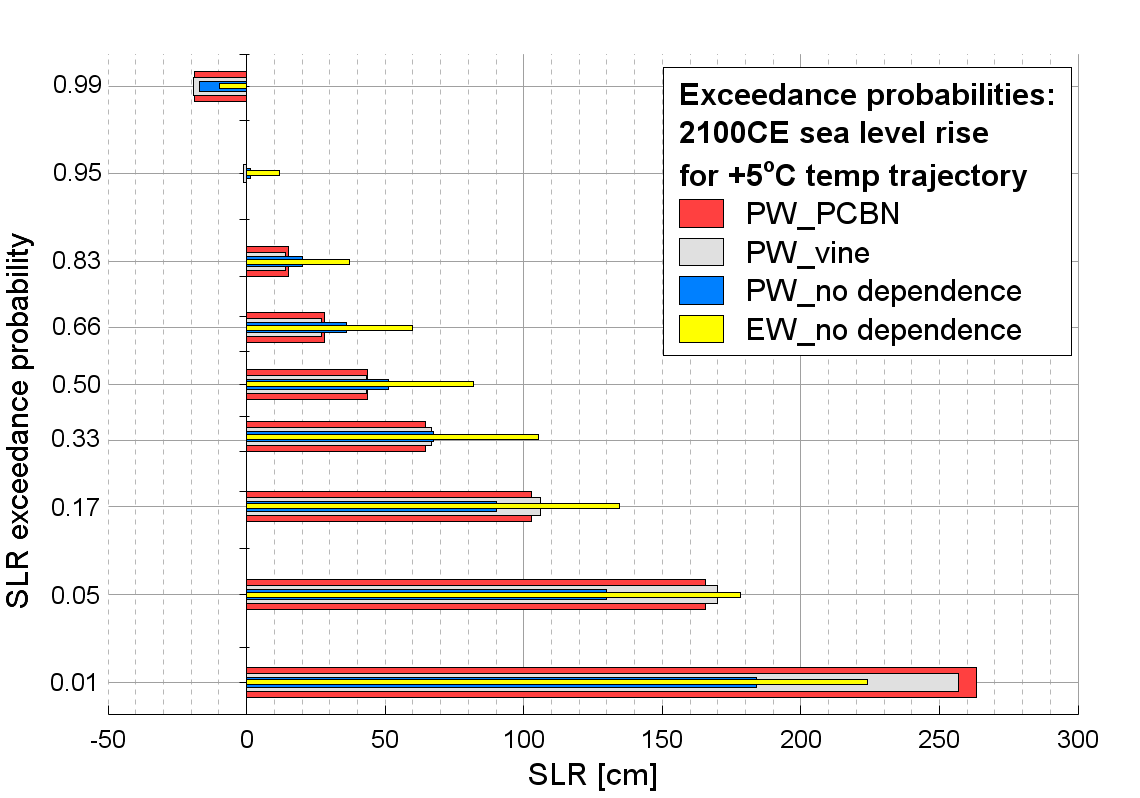

Supplement: Supplementary file 1 [file entropy-26-00949-s001.zip › Figures/Kurowicka SI Fig2.png]

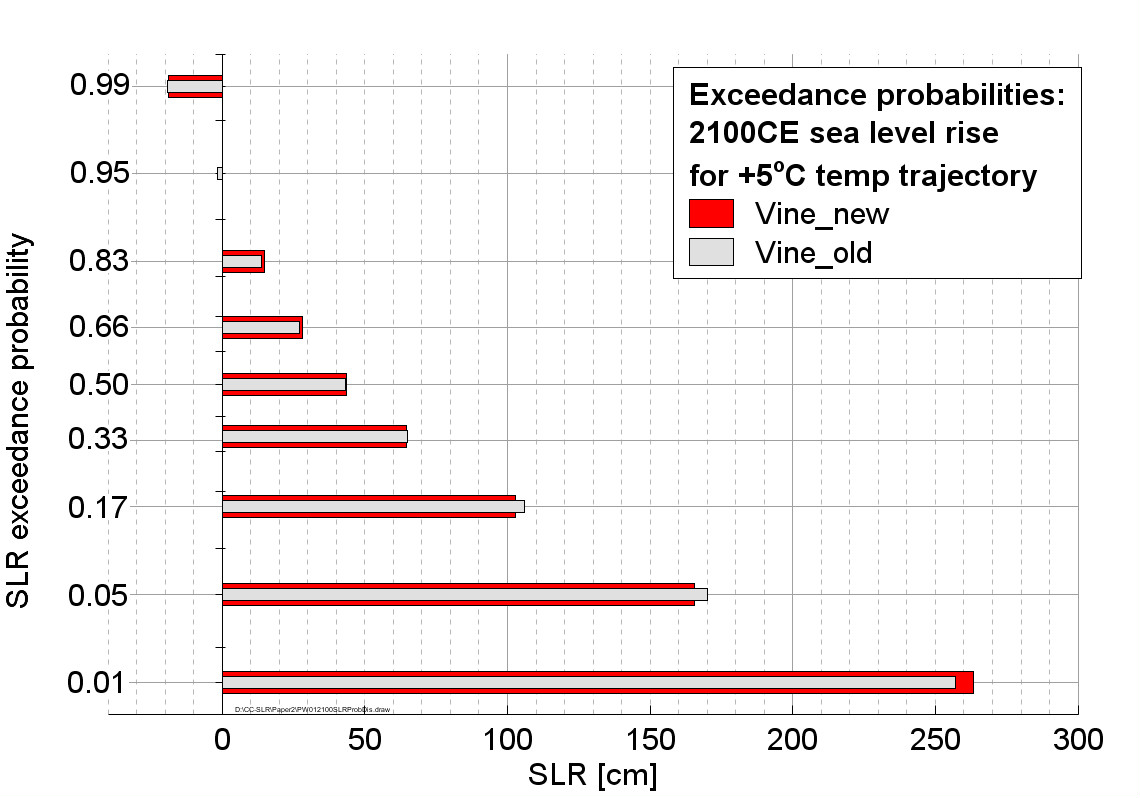

Supplement: Supplementary file 1 [file entropy-26-00949-s001.zip › Figures/PW012100SLRProbDis.jpg]

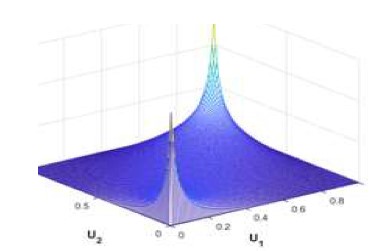

Supplement: Supplementary file 1 [file entropy-26-00949-s001.zip › Figures/Rew2Picture1.jpg]

Soln. ■ PW\_dep2 ■ PW\_dep1 ■ PW\_ind ■ EW\_ind

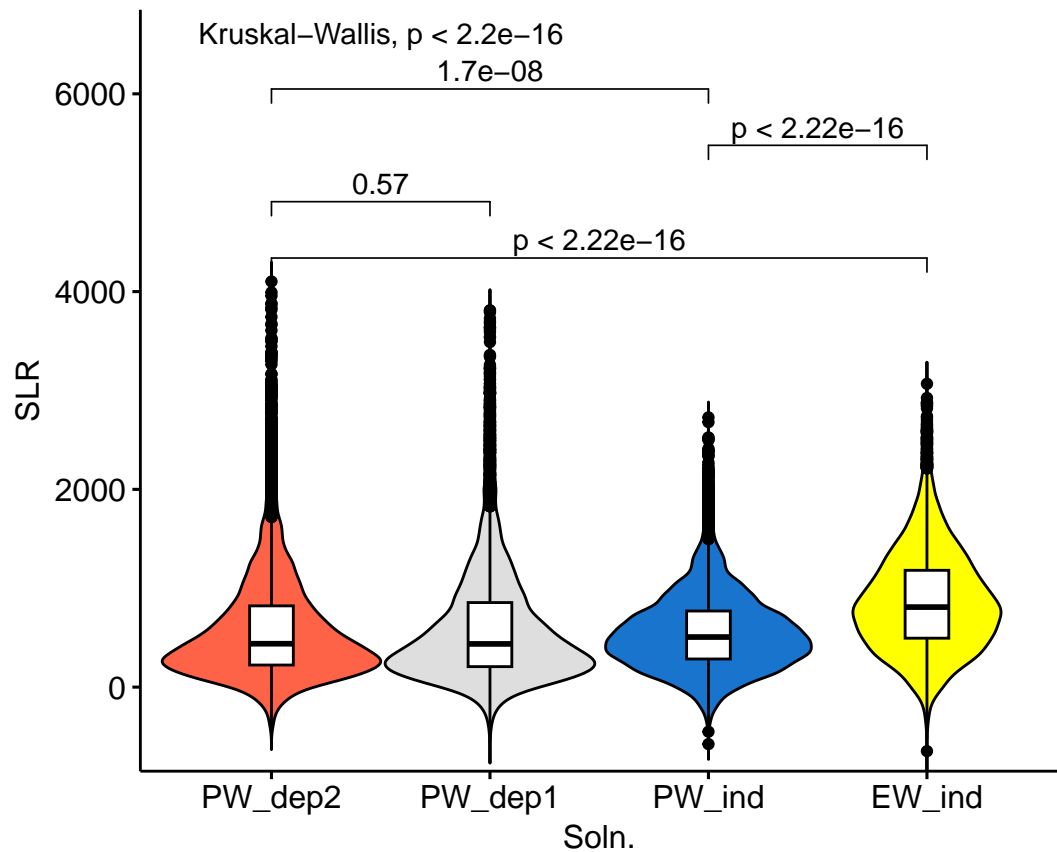

Supplement: Supplementary file 1 [file entropy-26-00949-s001.zip › Figures/SLRExcProbDis.pdf]

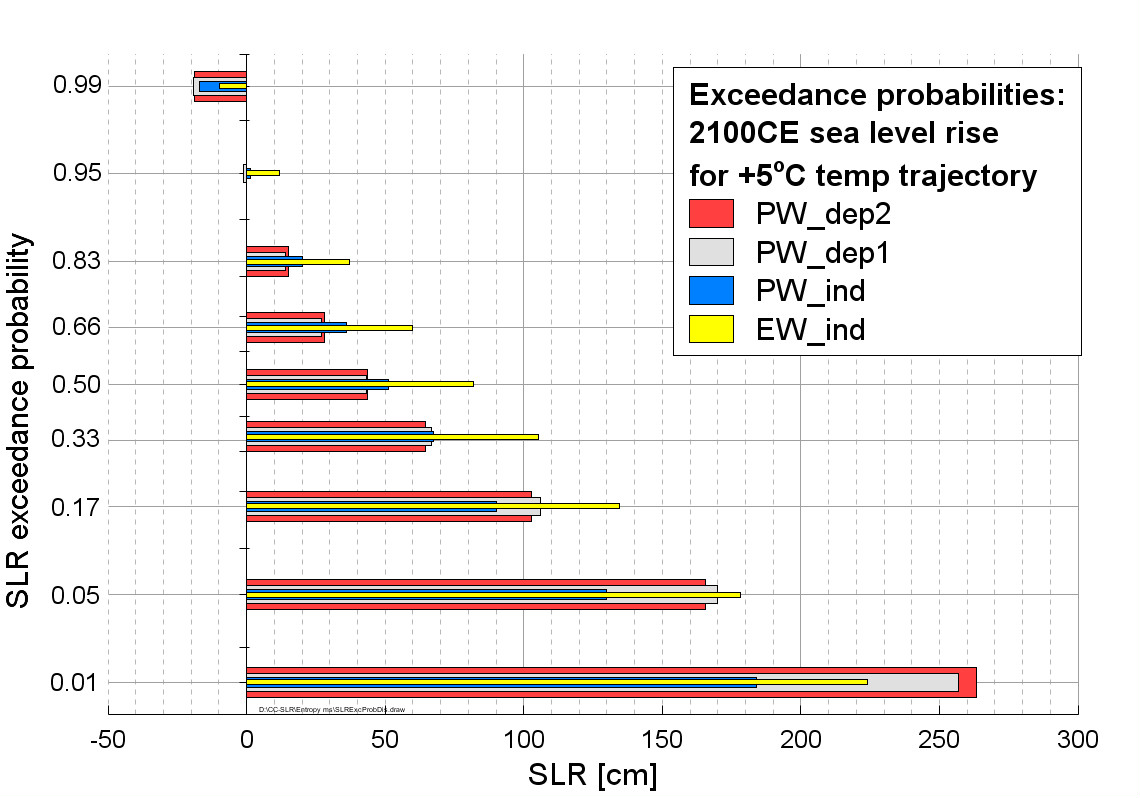

Supplement: Supplementary file 1 [file entropy-26-00949-s001.zip › Figures/SLRExcProbDis_alt weightings.jpg]
